# Low prevalence of epilepsy and onchocerciasis after more than 20 years of ivermectin treatment in the Imo River Basin in Nigeria

**DOI:** 10.1186/s40249-019-0517-9

**Published:** 2019-01-23

**Authors:** Joseph N. F. Siewe, Chinyere N. Ukaga, Ernest O. Nwazor, Murphy O. Nwoke, Modebelu C. Nwokeji, Blessing C. Onuoha, Simon O. Nwanjor, Joel Okeke, Kate Osahor, Lilian Chimechefulam, Ann I. Ogomaka, Augustine A. Amaechi, Chika I. Ezenwa, Monika N. Ezike, Chidimma Ikpeama, Ogechi Nwachukwu, Austine I. Eriama-Joseph, Berthram E. B. Nwoke, Robert Colebunders

**Affiliations:** 1 Global Health Institute, University of Antwerp, Campus Drie Eiken, Doornstraat 331, 2610 Wilrijk, Antwerp, Belgium; 2 Imo State University, Owerri, Nigeria; 3 Federal Medical Centre, Owerri, Nigeria; 4 Madonna University Teaching Hospital, Elele, Nigeria; 5 Imo State Ministry of Health, Owerri, Nigeria

**Keywords:** Epilepsy, Onchocerciasis, Nigeria, Ivermectin, Ov16

## Abstract

**Background:**

High epilepsy prevalence and incidence have been reported in areas with high onchocerciasis transmission. Recent findings suggest that proper community-directed treatment with ivermectin (CDTI) is potentially able to prevent onchocerciasis-associated epilepsy (OAE). We assessed the epilepsy prevalence and onchocerciasis transmission in two Nigerian villages following more than 20 years of CDTI.

**Methods:**

A cross-sectional door-to-door survey was performed in two villages in the Imo River Basin reported to be mesoendomic for onchocerciasis (Umuoparaodu and Umuezeala). Individuals were screened for epilepsy using a validated 5-item questionnaire. Persons suspected to have epilepsy were examined by a neurologist or a physician with training in epilepsy for confirmation. Onchocerciasis was investigated via skin snip microscopy and rapid diagnostic tests for Ov16 antibodies. Results were compared with previous findings from the Imo river basin.

**Results:**

A total of 843 individuals from 257 households in the two villages were encountered. We detected four persons with epilepsy (PWE) giving a crude epilepsy prevalence of 0.5%. This finding differs from observations reported 14 years ago which showed an epilepsy prevalence of 2.8% in the neighbouring village of Umulolo (*P* = 0.0001), and 1.2% from 13 villages in the Imo river basin (*P* = 0.07). The seroprevalence of Ov16 antibodies was found to be 0%. Only 4.6% of skin snips were positive compared to 26.8% in previous surveys (*P* < 0.0001). Ivermectin mass distribution coverage in the study sites in 2017 was 79.7%.

**Conclusions:**

A low epilepsy and onchocerciasis prevalence was observed following more than 20 years of CDTI in the Imo River Basin. Absence of Ov16 antibodies indicates minimal transmission of onchocerciasis. These results contrast with observations from areas of high onchocerciasis transmission, where epilepsy prevalence and incidence remain high. Findings from this study suggest that sustained efforts could eventually achieve elimination of onchocerciasis in these villages.

**Electronic supplementary material:**

The online version of this article (10.1186/s40249-019-0517-9) contains supplementary material, which is available to authorized users.

## Multilingual abstracts

Please see Additional file 1 for translations of the abstract into five official working languages of the United Nations.

## Background

Epilepsy is a chronic disease estimated to affect 50 million people worldwide according to World Health Organization (WHO) [1]. In general, higher prevalence and incidence of epilepsy are reported from those populations living in low and middle income countries (LMICs) when compared to industrialized countries; the former account for nearly 80% of the global epilepsy burden [1]. The etiology of epilepsy is very diverse and not yet fully understood. Besides perinatal insults and traumatic injury to the brain, infectious diseases can trigger epilepsy as well. Several parasitic infections have been associated with epilepsy such as cerebral malaria, toxoplasmosis, neurocysticercosis and onchocerciasis [2, 3]. A parasitic etiology of epilepsy implies that many cases could be prevented through timely anti-parasitic treatment.

An epidemiological association between epilepsy and onchocerciasis had already been evoked in Mexico in the 1930s [4] and in Africa (western Uganda) in the early 1990s [5, 6]. A case-control study performed in 1991–1992 in the Mbam Valley in Cameroon prior to community-directed treatment with ivermectin (CDTI) demonstrated a significantly higher microfilarial load in persons with epilepsy than in controls [7]. Study results from other onchocerciasis-endemic African countries underline this association [2, 8]. To describe this epidemiological phenomenon, the term onchocerciasis associated epilepsy (OAE) was proposed by Kaiser and colleagues [8]. More recently, diagnostic criteria for OAE were proposed [9]. In Tanzania, Uganda and South Sudan, a distinct form of epilepsy has been described as nodding syndrome (NS) [10, 11]; NS is a debilitating epileptic disorder mainly affecting children between the ages of 3 to 18 years [12]. Case-control studies in northern Uganda and South Sudan focusing on NS patients showed a higher prevalence of onchocerciasis in NS cases compared to non-epileptic controls [13, 14]. It is however unclear how onchocerciasis might cause NS. Johnson et al. suggested that an immunological cross-reaction of onchocerciasis-specific antibodies may provoke a neurotoxic reaction and trigger NS [15]. On the other hand, a cohort study in Cameroon showed a strong dose-response type of association between onchocercal skin microfilariae density as a child and the development of epilepsy later in life [16]. The latter study suggests that the microfilariae themselves are the trigger of the epilepsy. Another phenotypic entity known as the Nakalanga syndrome (epilepsy, stunting, absence or delayed development of external signs of sexual development) also occurs in onchocerciasis-endemic areas [17]. Head nodding seizures and Nakalanga features are increasingly considered as part of the OAE spectrum, alongside other forms of epilepsy [18].

In 2004, following a decade of annual community-directed treatment with ivermectin (CDTI) in the Imo River Basin in Nigeria, a high epilepsy prevalence was observed in villages with high onchocerciasis prevalence [19]. Since 2004, these villages have benefited from another decade of annual CDTI and four years of biannual CDTI. In northern Uganda it was observed that optimal onchocerciasis control was potentially able to stop the incidence of NS and other forms of OAE [20]. Therefore we sought to investigate the effect of more than two decades of CDTI on the prevalence of epilepsy in villages which hitherto were initially meso/hyperendemic for onchocerciasis [21]. We also verified ivermectin coverage and the ongoing transmission risk for onchocerciasis in the area.

## Methods

### Study setting

**Fig. 1:**
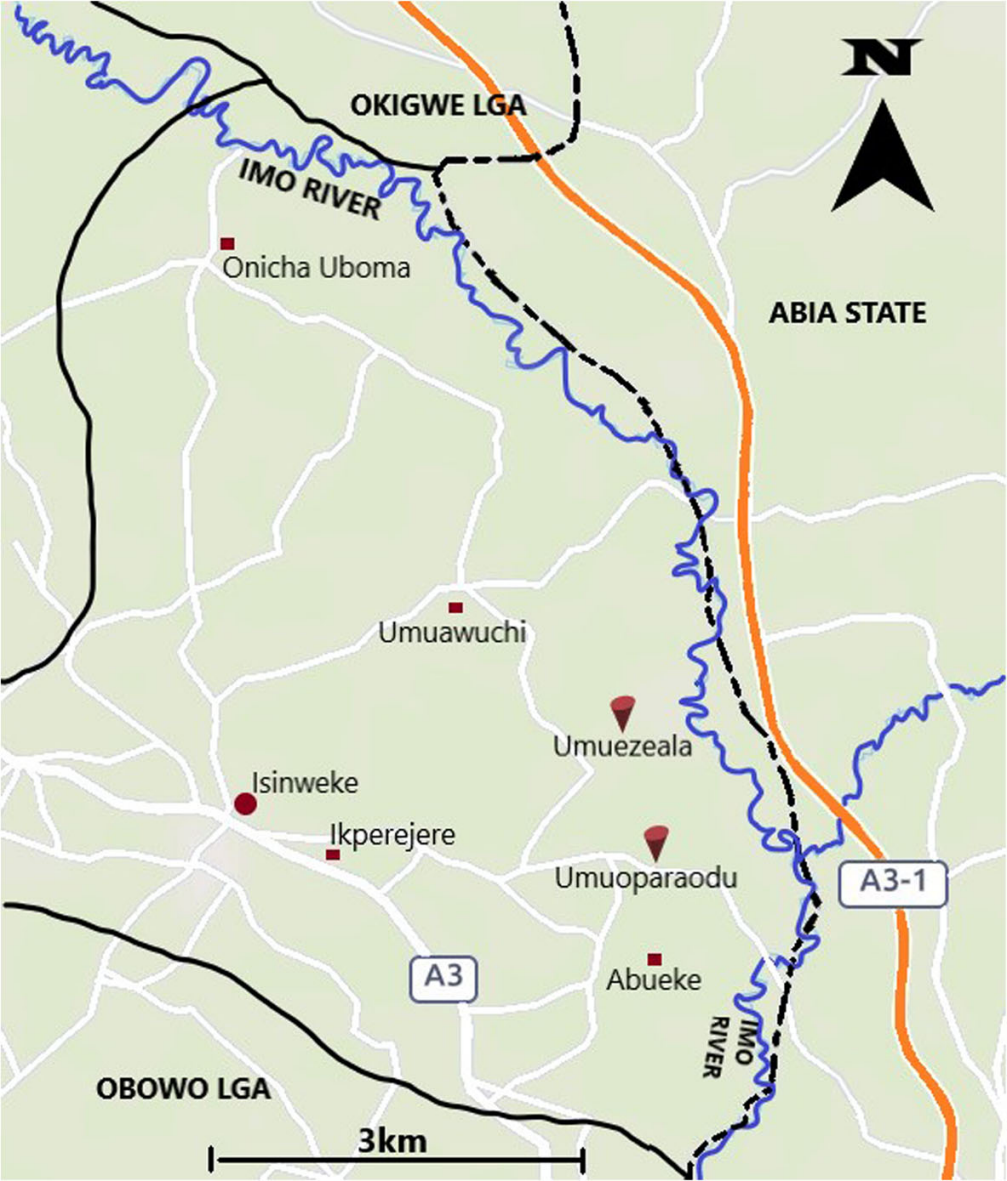
Map showing the locations of Umuezeala and Umuoparaodu. Distance from the Imo river: Umuezeala = 1.07 km; Umuoparaodu = 1.17 km

The study took place in June 2018 and focused on villages located in the Imo River Basin (Imo State, Nigeria), a known breeding site of the vector *Simulium damnosum* complex. We selected the Ihite Uboma Local Government Area (LGA), and the Abueke Autonomous Community. From Abueke, we selected the two neighbouring villages closest to the Imo River (Umuezeala and Umuoparaodu), so as to verify possible onchocerciasis transmission risks (Fig. 1). These villages are found within a 40 km radius of Umulolo village in the Okigwe LGA, which had the highest reported epilepsy prevalence during the previous survey in the Imo river basin [19]. Due to logistical reasons, we were not able to include Umulolo in our survey at this time.

### Study population

The entire population of the selected villages was included in the study. The dominant ethnic group in the area is Ibo. Subsistence farming and petty trading are the main occupations of the people.

### Study design

The study was designed as a community-based cross-sectional survey, following a two-stage approach for epilepsy case identification at village level. We used a door-to-door approach to identify epilepsy cases in the communities. For each village, the local authorities were seen a few weeks before the field survey. The study procedures were explained to them and their collaboration was obtained. Thereafter, sensitization sessions were organized in the villages to prepare the inhabitants for the study. The research team included a neurologist (EON), ten physicians including one with training in epilepsy (JNFS), parasitologists (CNU), laboratory technicians and public health specialists. We included the local healthcare workers (nurses) and community distributors of ivermectin because they were familiar with the populations and could fluently speak the local Ibo language. All team members were trained on how to do the household survey and ensure confidentiality. The questionnaire was pretested, and was administered primarily in the local language (Ibo) as most individuals did not understand English. In each village, community leaders were interviewed to obtain information pertaining to the villagers’ lifestyles, complaints about blackfly bites, their knowledge of onchocerciasis, epilepsy and the emigration/immigration trends over the years.

### Household survey

During the door-to-door survey, we proceeded by first obtaining the signed informed consent of the household head or his representative. The survey was done in two stages. In a first phase, household information was collected which included the ages and sexes of all household occupants, ivermectin treatment status, length of stay of the family in the village, main occupation, notion of previous death of a person with epilepsy and notion of any blind individual in the household. The second part of the family survey consisted of a validated 5-item questionnaire for epilepsy screening [22] which was administered to every member of the household. The five screening questions were: i) history of loss of consciousness with or without loss of bladder control and/or drooling; ii) history of absence or sudden lapse of consciousness of brief duration; iii) history of sudden involuntary jerks of the arms and/or legs (convulsions) lasting for a few minutes; iv) history of sudden and brief bodily sensations, seeing or hearing things that are not there, or smelling strange odours; and v) having been told that he/she had epilepsy or had experienced two epileptic episodes. Family survey data was noted on paper forms.

### Epilepsy confirmation

Anyone with a positive answer to any of the questions during the screening phase was considered as a person suspected to have epilepsy, and was further examined by the neurologist/physician with training in epilepsy. The neurological evaluation included both the medical history and a detailed neurological examination to confirm or reject the diagnosis of epilepsy. In case the epilepsy diagnosis was confirmed, the age at the onset of seizures, seizure types and frequencies were noted. Questions were asked concerning potential causes of epilepsy such as birth trauma, head injury, meningo-encephalitis, and cerebral malaria. Data collection by clinicians was done via tablets using the Open Data Kit (https://opendatakit.org). A data manager was responsible for guaranteeing the completeness and good quality of data, and to ensure data transfer from each tablet to the central server for data security. All confirmed cases of epilepsy were referred to the community health centre for proper follow-up.

For quality control, the neurologist (EON) and the physician trained in epilepsy (JNFS) participated in the household visits during the first day of the survey and were thus able to clerk healthy individuals in order to confirm that the latter did not have epilepsy.

### Ov16 rapid diagnostic tests

During the household visits, children aged 6–10 years were identified and referred with their parents/guardians to a workstation to be tested for Ov16 antibodies, using a drop of blood obtained by finger pricking. The children who reported to the workstation came from random areas of the villages and they were all tested. Additionally, persons with confirmed epilepsy and volunteering healthy adults were also tested. The kit used for the tests was the onchocerciasis IgG4 rapid diagnostic test (Ov16 RDT, Standard diagnostics, Inc., Giheung-gu, Yongin-si, Gyeonggi-do, Korea).

### Skin snip for onchocerciasis diagnosis

Participants tested for Ov16 antibodies were also skin snipped for a parasitological diagnosis of onchocerciasis. Two skin snips were obtained from each posterior iliac crest or scapular region using sterilized Holt-type punches. The snips were immediately placed in different wells of a microtitre plate containing three drops of normal saline solution and incubated for 24 h at room temperature to allow the microfilaria (mf) to emerge into the fluid. After the incubation period, each well was viewed by inverse microscopy using × 40 magnification and microfilariae counted in turns by a trained technician and a parasitologist.

### Definitions

A *case of epilepsy* was defined as a person who had experienced at least two seizures, unprovoked and without fever, with a minimal time difference of 24 h between the two events [23].

*New cases of epilepsy* were defined as cases that appeared within the last 12 months preceding the study period.

*Immigrant households* were defined as families which were not natives from the study site, but lived in the village.

*Onchocerciasis associated epilepsy* (OAE) was defined using previously published criteria [9] which included: residence in the village for at least three years, onset of epilepsy between 3 and 18 years, high prevalence of epilepsy in the village, no obvious cause for epilepsy obtained from the medical history and normal psychomotor development prior to the onset of seizures.

*Ivermectin coverage* referred to the percentage of the entire population that reported taking ivermectin in 2017. The objective was to attain at least 85% coverage, because it is considered that 15% of the population are composed of pregnant women, children under five, and the seriously ill who are ineligible for ivermectin treatment [24].

### Data management and analysis

Data was entered into Microsoft Excel 2016 (Microsoft Corporation, Washington, USA) and the Open Data Kit server (ODK Development Team, Washington, USA), and analysed using GraphPad Prism 5 (GraphPad Software, California, USA). For descriptive statistics, continuous data was expressed as means/standard deviations or median/interquartile range (IQR) as appropriate, while categorical data was expressed as percentages. The prevalence of epilepsy was computed by dividing the number of confirmed epilepsy cases by the total number of people registered in the households visited. Past and present prevalences for epilepsy and onchocerciasis were compared using the Chi-squared test at a significance level of 95%.

### Ethical considerations

The study was approved by the ethics committee of the University of Antwerp, Belgium and the ethical committee of the Imo State University, Owerri, Nigeria. All the household heads and individual subjects provided a written informed consent to participate in the study. Parents consented for Ov16 testing in children. In case of illiteracy, an informed verbal consent was given and a thumb print signature was obtained. Information was coded and made anonymous during data entry. The research team was trained to ensure confidential treatment of the data obtained.

## Results

### Study population

A total of 843 individuals were screened for epilepsy in 257 households from the two villages. Table 1 shows the characteristics of the individuals and households surveyed in June 2018. The median household size was 3 (interquartile range, IQR: 2–4). There was a masculine preponderance in the study population, with 52.4% of male participants versus 47.6% females and a median age of 36 years (IQR: 18–55). There were 13 immigrant households (5.1%) with a mean length of stay in the village of one year. Two blind persons (0.2%) were found in the study villages. Seven households (2.7%) reported that a family member with epilepsy died in the past at a median age of 19 years; only one person with epilepsy (0.1% of the population) died within the last year. No household reported that a family member developed epilepsy within the 12 months preceding our survey (incidence = 0). Farming and trading were the main activities, being practised by 64.0 and 10.9% of households respectively.

**Table 1:** Characteristics of the study population

	Umuezeala	Umuoparaodu	Overall
Households			
Total number of households	130	127	257
Native households: *n* (%)	124 (95.4%)	120 (94.5%)	243 (94.5%)
Immigrant households: *n* (%)	6 (4.6%)	7 (5.5%)	13 (5.1%)
Median household size: *n* (IQR)	3 (2–5)	3 (2–4)	3 (2–4)
Family history of death from epilepsy: *n* (%)	6 (4.6%)	1 (0.8%)	7 (2.7%)
Median age at death of PWE in years	19	19	19
Participants			
Number of individuals	465	378	843
Age: median (IQR)^a^	33 (19–55)	38 (18–55)	36 (18–55)
Age distribution: n (%)^a^			
0–9 years	52 (11.3%)	55 (14.6%)	107 (12.8%)
10–19 years	70 (15.2%)	49 (13.0%)	119 (14.2%)
20–29 years	79 (17.1%)	45 (12.0%)	124 (14.8%)
30–39 years	62 (13.5%)	48 (12.8%)	110 (13.1%)
40–49 years	55 (11.9%)	58 (15.4%)	113 (13.5%)
≥ 50 years	143 (31.0%)	121 (32.2%)	264 (31.5%)
Gender: *n* (%)^b^			
Female	233 (50.3%)	165 (44.2%)	398 (47.6%)
Male	230 (49.7%)	208 (55.8%)	438 (52.4%)

*IQR* Interquartile range, *PWE* Person(s) with epilepsy; ^a^6 missing data; ^b^7 missing data

### Prevalence of epilepsy

A total of 43 individuals answered positively to at least one epilepsy screening question, giving suspected epilepsy prevalence rates of 5.1%. Of these 43 persons suspected to have epilepsy, eight showed up at the consultation desk for confirmation, four were seen in their homes and for the remaining 31 who were unavailable at the time of the survey, the neurologist/physician trained in epilepsy obtained their medical history from family relatives. After thorough history taking for all suspected cases and neurological examination for the available participants, only four out of the 43 persons suspected to have epilepsy met the clinical description of epilepsy, giving a prevalence for confirmed epilepsy of 0.5%. Three (75%) of the four persons with confirmed epilepsy met some OAE criteria: their onset of seizures was between 3 and 18 years, there was no obvious cause for the epilepsy and their psychomotor development prior to the onset of seizures had been normal. They were all born and had lived in other onchocerciasis-endemic areas: one in Abuja, one in Katsina and the other in North West Cameroon. They only migrated into the study villages later. No nodding seizures were reported (Table 2) and no person with epilepsy (PWE) was stunted.

**Table 2:** Characteristics of confirmed persons with epilepsy

		Umuezeala	Umuoparaodu	Overall
Number of confirmed PWE		3	1	4
Prevalence of epilepsy		0.6%	0.3%	0.5%
Age of confirmed PWE in 2018: Median (IQR)		18 (16–45)	18	18 (16.5–38.3)
Age at onset of epilepsy: Median (IQR)		7 (6–30)	15	11 (6.3–26.3)
Duration of epilepsy in years		12 (9–15)	3	10.5 (4.5–14.3)
Seizure type	Generalized tonic clonic	3 (100%)	1 (100%)	4 (100%)
	Absences	1 (33.3%)	1 (100%)	2 (50%)
	Focal	0	0	0
	Nodding	0	0	0
Seizure frequency	Daily: > 30/month	0	0	0
	Monthly: > 12/year	3 (100%)	1 (100%)	4 (100%)
	Yearly: < 12/year	0	0	0
Burns / wounds		0	0	0
Past medical history	Birth dystocia	0	0	0
	Cerebral Malaria	0	0	0
	Measles	0	0	0
	Head trauma	0	0	0
	Probable eclampsia	1 (33.3%)	0	1 (25%)
Immigrants		2 (66.7%)	1 (100%)	3 (75%)
Clinical signs of onchocerciasis	Leopard skin	0	0	0
	Nodules	0	0	0
Seizure treatment	Antiepileptic drugs	3 (100%)	0	3 (75%)
	Traditional medicine	0	1 (100%)	1 (25%)
Ivermectin use prior to seizure onset		1 (33.3%)	0	1 (25%)
Positive Ov16 test		0	0	0
Positive skin snip		0	0	0

IQR: Interquartile Range; PWE: Person(s) with epilepsy

The 39 persons suspected to have epilepsy in whom the diagnosis was not confirmed had a median age of 43 years (IQR: 25–60) and had the following differential diagnoses: history of only one seizure, febrile seizures during childhood, non-epileptic tremor, dizziness, dysesthesia and cognitive symptoms such as reduced memory and awareness.

### Performance of the epilepsy screening tool

Of the 43 persons who were suspected to have epilepsy, 32 (74.4%) answered positively to question 1 of the questionnaire, 14 (32.6%) to question 2, 19 (44.2%) to question 3, 15 (34.9%) to question 4 and 14 (32.6%) to question 5. When using at least one positive answer to any of the five questions for screening, the positive predictive value for the five questions screening tool was 9.3% (4/43). When a combination of questions is used for screening, consisting of a positive answer to question 5 (highest specificity during validation [22]) and to any other question, the positive predictive value increased to 28.6% (4/14).

On the first day of the survey, the neurologist/physician trained in epilepsy visited 17 random households in which 51 individuals (6.0% of the study population) answered negatively to the five questions; all of them were clinically confirmed as not having epilepsy. On this limited sample, the negative predictive value for the 5-question screening tool was 100%. The sensitivity and specificity of the 5-item questionnaire for epilepsy screening in our study was 100 and 56.7% respectively (Table 3).

**Table 3:** Sensitivity and specificity of the 5 questions for epilepsy diagnosis

		Confirmed epilepsy	
		Yes	No
Positive screening	Yes	a = 4	b = 39
	No	c = 0	d = 51

Sample population: *N* = 94

Sensitivity: a/(a + c) = 100%

Specificity: d/(b + d) = 56.7%

Positive predictive value: a/(a + b) = 9.3%

Negative predictive value: d/(c + d) = 100%

### Onchocerciasis endemicity and CDTI coverage

All Ov16 tests from 87 tested participants who reported for testing (52 children aged 6–10 years and 35 volunteering adolescents/adults) were negative. Four of these 87 participants had positive skin snips (onchocerciasis prevalence: 4.6%; Median microfilarial load: 2 mf per skin snip [IQR: 1–3]); the age range of skin snip-positive participants was 6–10 years and none of them had ever taken ivermectin. Based on verbal reports of ivermectin use by participants in the households, we calculated the CDTI coverage for the year 2017 to be 79.7%.

### Comparison between previous and current survey

A survey of 13 villages in the Imo river basin in 2004 revealed an epilepsy prevalence of 1.2% and average skin snip positivity of 26.8% [19]. In 2018, the results of our investigation in two villages located in the same area and closest to the Imo river were compared with the 2004 findings. We observed a difference in onchocerciasis prevalence (26.8% vs 4.6%, *P* < 0.0001) and a non-significant difference in epilepsy prevalence (1.2% vs 0.5%, *P* = 0.07) (Table 4). When our findings were compared with the 2004 results from Umulolo Village alone [19] (situated within 40 km from our study site), differences were observed in the prevalence of both onchocerciasis (36.0% vs 4.6%, *P* < 0.0001) and epilepsy (2.8% vs 0.5%, *P* = 0.0001).

**Table 4:** Comparison between previous and present surveys

	Dozie et al. 2004 [19]	Siewe et al. 2018	Remarks
Study sites	13 villages in the Imo river basin, Nigeria	Two villages in the Imo river basin, Nigeria	The two villages in 2018 were those closest to the Imo river
Number of years of CDTI before the study	10 years	24 years	The 24 years of CDTI in 2018 included four years of biannual distribution
Methods	- Door-to-door epilepsy surveys - Epilepsy screening by asking about previous epilepsy experience in the households, and confirmation by a doctor trained in paediatric neurology - Skin snip microscopy, no Ov16 testing	- Door-to-door epilepsy surveys - Epilepsy screening using a 5-item questionnaire, and confirmation by a neurologist or a doctor trained in epilepsy - Skin snip microscopy and Ov16 testing	Possible underestimation of epilepsy in 2004 because only one screening question was asked, and it is likely that only generalized convulsive seizures were detected
Onchocerciasis prevalence	26.8%	4.6%	*P* < 0.0001
Microfilaria density	22.1 per mg of skin snip	2 per skin snip	Average density from all infected participants
Ov16 RDT positive results	Not done	0%	Children and adults were tested in 2018
Onchocerciasis endemicity	Meso-endemic	Hypo-endemic	Reduced endemicity after 14 years of CDTI
Epilepsy prevalence	1.2%	0.5%	*P* = 0.07

*CDTI* Community-directed treatment with ivermectin, *RDT* Rapid diagnostic test

### Community leaders’ opinions

Five community leaders were interviewed, two from Umuezeala and three from Umuoparaodu. The highlights of their responses were as follows: (i) Presently, epilepsy is not frequent in the village, and therefore not well known by the community. All five interviewees could recall at most one death due to epilepsy in recent years. (ii) Blindness is not common presently as compared to the past. They believed that ivermectin distribution helped to reduce blindness in the community. (iii) There are complaints of insects’ bites, mostly near the Imo river and in the farm. The nuisance due to insects has not changed with time. Villagers burned the chaffs from palm nuts so that the smoke should repel insects from their homes. However, they were still frequently bitten by insects while working in the farms. (iv) People do not rear pigs in the villages. (v) There had been more immigration than emigration in the community during the recent years.

## Discussion

For several decades, the Imo River Basin had been a known mesoendemic focus for onchocerciasis, which led to the implementation of annual CDTI in 1994 [19, 21] and biannual CDTI since 2015. Previous reports highlighted a high epilepsy prevalence in this area [19]. Our study in 2018 investigated the onchocerciasis and epilepsy situation after 24 years of CDTI and compared the results with observations made 14 years earlier (2004), when only 10 rounds of annual CDTI had been implemented [19]. The two villages under investigation were close to the Imo river and located within a 40 km radius of Umulolo, which has a similar ecological environment and had the highest epilepsy prevalence (2.8%) in the previous survey [19]. As in the 2004 study by Dozie et al. [19], we used the house-to-house approach which remains the standard for epilepsy surveys [25]. A lower epilepsy prevalence was observed when comparing our findings with those from Umulolo, alongside an almost inexistent transmission of onchocerciasis in 2018.

More than half of the village residents were older than 30 years; this population structure differs from typical African settings which generally have youthful populations [26]. Among other reasons, a possible explanation could be an important immigration rate of adults into the study villages as reported by community leaders. We also noted a low epilepsy-related mortality of 0.1% suggesting that the PWE are likely to live beyond the age of 30 years.

The fact that most PWE in the 2004 study were older than 20 years could be due to an age shift of PWE from 10 to 19 years to 20–29 years because of a reduced incidence of OAE after a decade of CDTI, as previously described [27]. In the present survey, three PWE (75%) were aged 10–19 years and met some of the OAE criteria. However, they were not born in the study sites and had not received ivermectin prior to seizure onset. They could have been exposed to onchocerciasis in their previous residences and later developed OAE-related seizures either before or after their immigration into the study villages. It is highly probable that effective onchocerciasis control has prevented children from developing epilepsy in the native population. The only PWE in our study whose seizures began in adulthood (at the age of 30 years) was a native of Umuezeala who had been receiving ivermectin and had a history of probable eclampsia followed by a non-traumatic coma a few months before the onset of seizures. In addition, the 0% seroprevalence of Ov16 antibodies coupled with a very low onchocerciasis prevalence reflect minimal onchocerciasis transmission resulting from optimal control measures.

Four (4.6%) out of 87 participants had positive skin snips but negative Ov16 results. This can be explained by the moderate sensitivity of the rapid test, especially with the very low microfilaria load observed. Indeed, Golden et al. reported up to 11% (27/248) false negatives when using the Ov16 RDT under laboratory conditions [28].

The sensitivity (100%) and specificity (56.7%) of the 5-question epilepsy screening tool were similar to the values reported during the validation of the questionnaire (95.1 and 65.6%, respectively) [22]. The positive predictive value was very low because of the low prevalence of epilepsy and possibly the fact that the community had little knowledge about epilepsy. The household participants readily attributed any unusual feeling to epilepsy, and may have answered yes to some of the screening questions even in the absence of seizures. The persons with confirmed epilepsy had a median age of 18 years as compared to 43 years for the persons in whom the diagnosis was suspected but later rejected; the latter presented mostly with non-epilepsy symptoms including signs of psychic senescence and parkinsonism that were most likely related to ageing [29]. We were able to improve the positive predictive value from 9.3 to 28.6% by using a combination of positive answers for epilepsy screening rather than just one positive answer.

The observed epilepsy prevalence of 0.50% is similar to the 0.53% reported in other parts of Nigeria [30] but different from previous findings in the Imo River Basin, and in particular Umulolo Village [19]. Lower epilepsy prevalence and transmission of onchocerciasis in 2018 compared to the 2004 survey, suggests that proper onchocerciasis control may be able to modify the epidemiology of epilepsy. Furthermore, the observed low epilepsy prevalence in villages having low onchocerciasis transmission contrasts with high epilepsy prevalence in meso/hyperendemic areas and further strengthens the hypothesis of an association between the two pathologies.

The responses from the community leaders suggest that blackflies still constitute a nuisance for the population. Despite the presence of the vector, onchocerciasis prevalence and transmission has reduced because proper CDTI has probably rendered their bites non-infective. This scenario differs from what happened in ivermectin-naïve onchocerciasis-endemic villages in the Democratic Republic of Congo, where a decreased transmission of onchocerciasis is thought to result from a progressive reduction of the number of blackflies [31].

Clinically, the main seizure types were generalized seizures (100% of cases) and absences (50% of cases). These results differ from those obtained by Osuntokun et al. in Western Nigeria in the 1980s, where a majority of focal seizures were found [30]. In our study, although 75% of PWE took antiepileptic drugs and 25% resorted to traditional medicine, all cases still experienced seizures on a monthly basis. Proper care of PWE in these remote villages must be instituted, and this necessitates the training of local non-physician staff in epilepsy care [32].

The strength of this study resides in the community-based methodology which makes the results comparable with previous findings. The use of Ov16 rapid tests in assessing transmission is a novel approach which was shown to be feasible and effective. As major limitation, not all cases were physically present for epilepsy confirmation; but given the advanced age of these suspected cases and with the concise description of their symptoms obtained from family relatives, it is unlikely that they had epilepsy. Finally, we were unable to exclude other causes of epilepsy including neurocysticercosis. But given that pigs were not reared in these communities, it is unlikely that neurocysticercosis is a confounder in the present survey.

## Conclusions

Optimal CDTI in the Imo River Basin has resulted in less onchocerciasis transmission with a concomitant decrease in epilepsy prevalence and incidence. Sustained efforts towards onchocerciasis elimination could potentially affect the epidemiology of epilepsy, as hypothesized by Dozie et al. [19]. Our findings further strengthen the possibility of a causal relationship between onchocerciasis and epilepsy.

## Additional file

Additional file 1: Multilingual abstracts in the five official working languages of the United Nations. (PDF 344 kb)

## Abbreviations

CDTI: Community-directed treatment with ivermectin
IgG4: Immunoglobulin G4 (antibody)
Mf: Microfilariae
OAE: Onchocerciasis associated epilepsy
ODK: Open data kit
PWE: Person(s) with epilepsy
RDT: Rapid diagnostic test
