# Supplementary material for: Low prevalence of epilepsy and onchocerciasis after more than 20 years of ivermectin treatment in the Imo River Basin in Nigeria
Source: Infect Dis Poverty. 2019 Jan 23;8:8. doi: 10.1186/s40249-019-0517-9 (PMC6343278; doi:10.1186/s40249-019-0517-9)
Supplement: Supplementary file 1 — Multilingual abstracts in the five official working languages of the United Nations. (PDF 344 kb) [file 40249_2019_517_MOESM1_ESM.pdf]

Translation of the abstract into the five official working languages of the United Nations

انخفاض معدل انتشار داء الصرع وداء كلابية الذنب بعد أكثر من 20 عامًا من العلاج بالأيفرمكتين في حوض نهر إيمو في نيجيريا

J. N. F. Siewe, C. N. Ukaga, E. O. Nwazor, M. O. Nwoke, M. C. Nwokeji, B. C. Onuoha, S. O. Nwanjor, J. Okeke, K. Osahor, L. Chimechefulam, A. I. Ogomaka, A. A. Amaechi, C. I. Ezenwa, M. N. Ezike, C. Ikpeama, O. Nwachukwu, A. I. Eriama-Joseph, B. E. B. Nwoke, R. Colebunders

الملخص
المقدمة: تم الإبلاغ عن ارتفاع معدل انتشار الصرع وحدوثه في المناطق التي ينتقل فيها داء كلابية الذنب. وتشير النتائج الحديثة إلى أنه من المحتمل أن العلاج المناسب الموجه محلياً بالأيفرمكتين (مكافحة داء العمى النهري من خلال العلاج المجتمعي الموجه بالإيفرمكتين) لديه القدرة على التغلب على الصرع المصاحب لداء كلابية الذنب (OAE). وقد قمنا بتقييم انتشار الصرع وداء كلابية الذنب في قريتين نيجيريتين بعد أكثر من 20 عامًا من استخدام العلاج الموجه محلياً بالأيفرمكتين (مكافحة داء العمى النهري من خلال العلاج المجتمعي الموجه بالإيفرمكتين).
الطرق: أجريت دراسة مقطعية مستعرضة من الباب إلى الباب في قريتين في حوض نهر إيمو ، ذُكر أنهما وسيط مستوطن لداء كلابية الذنب (أوموباراودي و أوميوزيلا). وتم فحص الأفراد بحثًا عن الصرع باستخدام استبيان مكون من 5 عناصر قد تم التحقق من صحته. تم فحص الأشخاص المشتبه في إصابتهم بالصرع من قبل طبيب أعصاب أو طبيب متدرب على مرض الصرع للتأكيد. تم فحص داء كلابية الذنب (العمى النهري) عبر الفحص المجهري للجلد والاختبارات التشخيصية السريعة للأجسام المضادة للدودة كلابية الذنب المتلوية Ov16. وتمت مقارنة النتائج مع النتائج السابقة من حوض نهرإيمو بنيجيريا.
النتائج: تم حصر إجمالي 843 فردًا من 257 أسرة في القريتين. وقد اكتشفنا وجود أربعة أشخاص مصابين بالصرع مما يؤدي لنسبة انتشار في مرض الصرع بنسبة تصل إلى 0.5%. وتختلف هذه النتيجة عن الملاحظات التي رُصِدت قبل 14 عاماً والتي أظهرت انتشار الصرع بنسبة 2.8% في قرية أومولو المجاورة (P = 0.0001)، و 1.2%في 13 قرية في حوض نهر إيمو(P = 0.07). وقد نزح الثلاثة الذين أصيبوا بالصرع بين سن 3-18 سنوات إلى موقع الدراسة. ووُجد أن الانتشار المصلي للأجسام المضادة للدودة كلابية الذنب المتلوية Ov16 نسبته تكاد تنعدم لنسبة 0%. ونسبة 4.6% فقط من العينات الجلدية كانت إيجابية مقارنة بـ 26.8% من الدراسات الاستقصائية السابقة(P < 0.0001). وقد تم توزيع ايفرمكتين لتغطية نطاق واسع في مواقع الدراسة عام 2017 بنسبة 79.7%.
الاستنتاجات: لوحظ انخفاض انتشار الصرع وداء كلابية الذنب بعد أكثر من 20 عامًا من مكافحة داء العمى النهري من خلال العلاج المجتمعي الموجه بالأيفرمكتين في حوض نهر إيمو. كذلك يشير غياب الأجسام المضادة للدودة كلابية الذنب المتلوية Ov16 إلى انخفاض انتقال داء كلابية الذنب. علاوة على ذلك تتعارض هذه النتائج مع الملاحظات من المناطق التي ينتشر فيها داء كلابية الذنب بنسبة كبيرة، حيث لا يزال انتشار الصرع وحدوثه بنسبة عالية هناك. وتشير النتائج التي توصلت إليها هذه الدراسة إلى أن الجهود المتواصلة يمكن أن تؤدي في نهاية المطاف إلى القضاء على داء كلابية الذنب في هذه القرى.

Translated from English version into Arabic by Nirmin Hashoum and Shymaa Yakot, through

TRANSLATORS
WITHOUT BORDERS

20 年以上伊维菌素治疗大幅降低尼日利亚 Imo 河流域癫痫和盘尾丝虫病患病率

Joseph N. F. Siewe, Chinyere N. Ukaga, Ernest O. Nwazor, Murphy O. Nwoke, Modebelu C. Nwokeji, Blessing C. Onuoha, Simon O. Nwanjor, Joel Okeke, Kate Osahor, Lilian Chimechefulam,

Ann I. Ogomaka, Augustine A. Amaechi, Chika I. Ezenwa, Monika N. Ezike, Chidimma Ikpeama, Ogechi Nwachukwu, Austine I. Eriama-Joseph, Berthram E. B. Nwoke and Robert Colebunders

摘要

引言： 盘尾丝虫病高传播地区的癫痫发病率和患病率较高。最近的研究发现，社区正确指导使用伊维菌素进行治疗有望预防盘尾丝虫病相关的癫痫。在尼日利亚两个村庄开展伊维菌素治疗 20 多年后，本研究对其癫痫患病率与盘尾丝虫病传播情况进行了评估。

方法： 本研究在 Imo 河流域的 Umuoparaodu 村和 Umuezeala 村开展挨家挨户的横断面调查，这两个村均为盘尾丝虫病中度流行区。通过标准的五项问卷进行癫痫筛查。疑似病例由一位受过癫痫确诊培训的神经科医生或内科医生进行检查。通过皮片镜检和基于 Ov16 抗体的快速检测对盘尾丝虫病例进行筛查。最后将该调查结果与以往结果进行比较。

结果： 本研究共纳入两个村庄的 257 户 843 个调查对象，发现 4 例癫痫患者，粗患病率为 0.5%。这与十四年前邻村 Umulolo 的癫痫患病率（2.8%）有显著差异（$P = 0.0001$），当时 Imo 河流域 13 个村庄癫痫的患病率为 1.2%（$P = 0.07$）。其中有 3 位 3–18 岁的患者，他们是后来移居这两个村的（癫痫发作前未注射疫苗）。Ov16 抗体检测的血清阳性率为 0%。皮片检测阳性率仅 4.6%，与之前 26.8%的阳性率具有显著差异（$P < 0.0001$）。2017 年这两个村庄的伊维菌素覆盖率达到 79.7%。

结论：尼日利亚 Imo 河流域开展伊维菌素社区指导治疗 20 年多年来，癫痫和盘尾丝虫病患病率保持在极低水平。Ov16 抗体检测阳性率为零说明盘尾丝虫病的流行率极低。与该研究结果相比，盘尾丝虫病高传播地区的癫痫患病率和发病率仍然很高。本研究表明，持久干预有助于最终消除盘尾丝虫病。

Translated from English version into Chinese by Cong-Shan Liu, edited by Jin Chen

CHINESE CENTER FOR DISEASE CONTROL AND PREVENTION
NATIONAL INSTITUTE OF PARASITIC DISEASES

Faible taux de prévalences de l'épilepsie et de l'onchocercose dans le bassin de la rivière Imo au Nigéria, après plus de 20 ans de traitement à l'ivermectine.

J. N. F. Siewe, C. N. Ukaga, E. O. Nwazor, M. O. Nwoke, M. C. Nwokeji, B. C. Onuoha, S. O. Nwanjor, J. Okeke, K. Osahor, L. Chimechefulam, A. I. Ogomaka, A. A. Amaechi, C. I. Ezenwa, M. N. Ezike, C. Ikpeama, O. Nwachukwu, A. I. Eriama-Joseph, B. E. B. Nwoke, et R. Colebunders

Résumé

Introduction : Une forte prévalence et une incidence élevée de l'épilepsie ont été signalées dans les régions où sévit une forte transmission de l'onchocercose. Des études récentes indiquent qu'un traitement approprié à l'ivermectine sous directives communautaires (TIDC) pourrait potentiellement prévenir l'épilepsie associée à l'onchocercose (EAO). Nous avons évalué la prévalence de l'épilepsie et la transmission de l'onchocercose dans deux villages nigérians après plus de 20 ans de traitement à l'ivermectine sous directives communautaires (TIDC).

Méthodes : Une enquête transversale porte-à-porte a été réalisée dans deux villages du bassin de la rivière Imo, signalés comme étant hyper endémiques à l'onchocercose (Umuoparaodu et Umuezeala). Des tests de dépistage de l'épilepsie ont été effectués chez des individus au moyen

d'une enquête validée, de cinq questions. Les personnes suspectées d'être atteintes d'épilepsie ont été examinées par un neurologue ou un médecin ayant suivi une formation concernant l'épilepsie. L'onchocercose a été recherché par microscopie par voie cutanée et par des tests de diagnostic rapides visant à déceler les anticorps Ov16. Les résultats ont été comparés à ceux qu'on avait trouvé précédemment dans le bassin de la rivière Imo.

Résultats: 843 personnes issues de 257 foyers dans les deux villages ont été approchées. Nous avons détecté cinq personnes atteintes d'épilepsie (révélant un taux brut de prévalence de personnes atteintes d'épilepsie (PWE) de 0.5%. Ce résultat est différent de ceux qui avaient été rapportés quatorze ans plus tôt et qui révélaient un taux de prévalence d'épilepsie de 2.8% dans le village voisin de Umololo (*P* = 0.0001), et de 1.2% dans 13 villages dans le bassin de la rivière Imo (*P* = 0.07). Les trois personnes atteintes d'épilepsie qui ont développé la maladie entre 3 et 18 ans avaient été dirigés vers le site d'étude. La séroprévalence des anticorps Ov16 s'est révélée être de 0%. Seulement 4.6% des prélèvements de peau ont été positifs comparés au taux de pourcentage des études précédentes qui était de 2.8% ($P < 0.0001$). La couverture de distribution de masse de l'ivermectine sur les sites d'étude en 2017 a été de 79.7%.

Conclusions: Une faible prévalence de l'épilepsie et de l'onchocercose a été observée après plus de 20 ans de traitement à l'ivermectine sous directives communautaires (CDTI) dans le bassin de la rivière Imo. L'absence d'anticorps Ov16 indique une faible transmission de l'onchocercose. Ces résultats contrastent avec ceux observés dans les régions ou sévit une forte transmission a l'onchocercose, et où les taux de prévalence et d'incidence restent élevés. Les résultats de cette étude indiquent que des efforts soutenus pourraient éventuellement mener à l'élimination de l'onchocercose dans ces villages.

Translated from English version into French by Iris Soliman and Isabelle Redon, through

TRANSLATORS
WITHOUT BORDERS

Низкие показатели распространенности эпилепсии и онхоцеркоза в бассейне реки Имо (Нигерия) по истечении более 20 лет лечения ивермектином

Дж. Н. Ф. Сиуе, С. Н. Укага, Е. О. Нвазор, М. О. Нвоке, М. С. Нвокеджи, Б. С. Онуоха, С. О. Нванджор, Дж. Океке, К. Осахор, Л. Шиншефулам, А. И. Огомака, А. А. Амаэчи, С. И. Эзенва, М. Н. Эзике, С. Икпеама, О. Нвачукву, А. И. Эриама-Джосеф, Б. Е. Нвоке и Р. Колебандерс.

Аннотация

Введение: Были получены данные о высоких показателях заболеваемости эпилепсией в районах, где распространен онхоцеркоз. По данным последних исследований правильное лечение местного населения ивермектином (ЛМНИ), вероятно, способно препятствовать распространению эпилепсии, связанной с онхоцеркозом (ЭСО). Мы определили показатели

распространенности эпилепсии и заболеваемости онхоцеркозом в двух нигерийских деревнях по истечении более 20 лет ЛМНИ.

Методы: В двух деревнях, расположенных в бассейне реки Имо, где были зарегистрированы случаи онхоцеркоза (Умуопараоду и Умуезеала), было проведено перекрестное исследование путем очного опроса населения. Диагностика эпилепсии проводилась с использованием утвержденного вопросника из пяти пунктов. Лица с подозрением на эпилепсию были осмотрены компетентным неврологом или терапевтом для подтверждения диагноза. Диагностика онхоцеркоза проводилась с помощью микроскопического исследования образцов кожи и экспресс-тестов на антитела к Ov16. Результаты были сопоставлены с ранее собранными в бассейне реки Имо данными.

Результаты: Всего в двух деревнях было опрошено 843 человека из 257 семей. Мы обнаружили четыре лица с эпилепсией (ЛСЭ), то есть общий коэффициент распространенности эпилепсии составил 0,5%. Данный результат отличается от результатов исследований, которые проводились 14 лет назад, - коэффициент распространенности эпилепсии в соседней деревне Умулоло составлял 2,8% ($P = 0{,}0001$), а в 13 деревнях в бассейне реки Имо – 1,2% ($P = 0{,}07$). Трое ЛСЭ, у которых эпилепсия развилась в возрасте 3-18 лет, переехали в район проведения исследования. Положительная серологическая реакция на антитела к Ov16 составила 0%. В результате исследования образцов кожи онхоцеркоз был обнаружен всего в 4,6%; по результатам предыдущих исследований этот показатель составил 26,8% ($P < 0{,}0001$). В 2017 г. массовое распространение ивермектина охватывало 79,7% площади района проведения исследований.

Выводы: Спустя более 20 лет ЛМНИ в бассейне реки Имо выявлены низкие показатели распространенности эпилепсии и онхоцеркоза. Отсутствие антител к Ov16 свидетельствует о низкой вероятности заражения онхоцеркозом. Эти результаты существенно отличаются от результатов исследований, которые проводились в районах с высоким показателем инфицирования онхоцеркозом, где показатель распространенности эпилепсии по-прежнему высок. Результаты настоящего исследования свидетельствуют, что при последовательном применении соответствующих мер в конечном итоге можно добиться ликвидации онхоцеркоза в этих деревнях.

Translated from English version into Russian by Daria and Liudmila Tomanek, through

TRANSLATORS
WITHOUT BORDERS

Baja prevalencia de epilepsia y oncocercosis después de más de 20 años de tratamiento con ivermectina en la cuenca del río Imo, en Nigeria

J. N. F. Siewe, C. N. Ukaga, E. O. Nwazor, M. O. Nwoke, M. C. Nwokeji, B. C. Onuoha, S. O. Nwanjor, J. Okeke, K. Osahor, L. Chimechefulam, A. I. Ogomaka, A. A. Amaechi, C. I. Ezenwa, M. N. Ezike, C. Ikpeama, O. Nwachukwu, A. I. Eriama-Joseph, B. E. B. Nwoke, y R. Colebunders

Resumen

Introducción: Se ha informado una elevada prevalencia e incidencia de epilepsia en áreas con alta transmisión de oncocercosis. Los hallazgos más recientes sugieren que un tratamiento adecuado con ivermectina dirigido a la comunidad puede prevenir la epilepsia asociada a la oncocercosis (OAE). Hemos evaluado la prevalencia de la epilepsia y la transmisión de la oncocercosis en dos aldeas nigerianas, luego de más de 20 años de tratamiento.

Metodología: Se realizó una encuesta transversal de puerta en puerta en dos aldeas en la cuenca del río Imo, consideradas mesoendémicas de oncocercosis (Umuoparaodu y Umuezeala). Se monitoreó la epilepsia en los individuos por medio de un cuestionario validado de cinco puntos. Las personas sospechosas de padecer epilepsia fueron examinadas por un neurólogo o un médico con formación en epilepsia para confirmar el diagnóstico. La oncocercosis se investigó mediante microscopia cutánea y pruebas diagnósticas rápidas para anticuerpos contra Ov16. Los resultados se compararon con hallazgos anteriores de la orilla del río Imo.

Resultados: Se encontró un total de 843 individuos de 257 casas en ambos poblados. Detectamos a cuatro personas con epilepsia lo que da una prevalencia de epilepsia bruta del 0,5 %. Este hallazgo difiere de las observaciones reportadas hace 14 años, que mostraron una prevalencia de epilepsia del 2,8 % en la aldea vecina de Umulolo ($P = 0.0001$), y de 1,2 % de 13 aldeas en la cuenca del Imo ($P = 0.07$). Las tres personas que habían desarrollado epilepsia entre los 3 y los 18 años de edad habían emigrado al sitio de estudio. Se descubrió que la seroprevalencia de los anticuerpos Ov16 era del 0 %. Solo el 4,6 % de las biopsias fueron positivas en comparación con el 26,8 % en encuestas anteriores ($P < 0.0001$) La cobertura de distribución masiva de ivermectina en los lugares de estudio en 2017 fue del 79,7 %.

Conclusiones: Se observó una baja prevalencia de epilepsia y oncocercosis después de más de 20 años de tratamiento en la cuenca del río Imo. La ausencia de anticuerpos Ov16 indica una mínima transmisión de oncocercosis. Estos resultados contrastan con las observaciones en áreas de alta transmisión de la oncocercosis, donde la prevalencia y la incidencia de la epilepsia siguen siendo altas. Los hallazgos de este estudio sugieren que los esfuerzos sostenidos podrían lograr realmente eliminar la oncocercosis de estos pueblos.

Translated from English version into Spanish by Raquel Hurtado and Lidia Norese, through

TRANSLATORS
WITHOUT BORDERS
